# Mpox and stigma

**DOI:** 10.2471/BLT.24.021224

**Published:** 2024-12-01

## Abstract

How do you effectively formulate and target explicit information about mpox exposure risk without driving stigmatization? Gary Humphreys reports.

“One of the big challenges we see is getting our clients to walk into a health facility to get the health services they are entitled to,” says Aimée Nshombo Furaha.

Director-general of Umande, a female sex worker advocacy group comprised of 14 sex worker solidarity committees, Furaha is based in Bukavu in South Kivu, a focal point for the mpox outbreak in the Democratic Republic of the Congo.

As of mid-November, over 39 501 suspected cases of mpox had been reported in this country, making it by far the highest burden country in the World Health Organization (WHO) African Region.

Mpox can be transmitted through contact with contaminated surfaces and close contact between people, including sexual contact.

In the surging outbreak in Africa, sex workers and their clients are considered key populations at risk. “One of the reasons Bukavu has become a hotspot for mpox transmission is the presence of miners and truckers,” Furaha explains. “Mining centres around Bukavu attract large numbers of sex workers and truck drivers who frequently cross the borders with Rwanda and Burundi in both directions.”

According to Furaha, many of the sex workers she deals with are young women fighting for survival. “They can have many clients a day, and often work unprotected because they do not have condoms or because clients pay more for unprotected sex,” she says.

Given the risks they face, why don’t more sex workers go to the clinic?

One answer is stigmatization. Defined as the negative labelling or discrimination of individuals or groups based on certain characteristics, conditions or behaviours, and leading to social exclusion and marginalization, stigmatization has played a significant role in recent mpox outbreaks, influencing both public health responses and the behaviours of affected populations.

In the case of the women that Furaha represents, relentless mistreatment and abuse at the hands of their clients, the police and health professionals discourages them from seeking treatment. “They are afraid to come forward,” Furaha says.

“We realized early on in the global mpox epidemic that began in 2022 that stigma was going to be an issue,” says Rosamund Lewis, WHO technical lead for the global mpox response.

WHO responded by releasing *Public health advice on understanding, preventing, and addressing stigma and discrimination related to mpox *in December 2022. The guidance outlines the potential impacts of stigma, and offers recommended language and actions to combat stigmatizing attitudes, discriminatory behaviours and policies.

“There is clearly a challenge here.”

Rosamund Lewis

Getting the language right was considered crucial, and in developing it WHO drew on lessons learned in responding to the human immunodeficiency virus (HIV) pandemic.

“There were so many parallels, including the fact that the disease disproportionately affected men who have sex with men who have multiple partners, many of whom were reluctant to seek testing or treatment due to the stigma around their sexual orientation,” says Meg Doherty, director of the WHO Department of Global HIV, Hepatitis and Sexually Transmitted Infections.

The term men who have sex with men (MSM) itself derives from those lessons learned. “It allowed health experts to address risk factors for sexually transmitted infections without making assumptions about sexual orientation and, just as importantly, allowed for targeted interventions based on sexual behaviour rather than identity, which is critical in reducing stigma and ensuring that health messaging reaches all relevant populations,” Doherty says.

The term sex worker is another de-stigmatized term, as is mpox, which was coined in 2022 by WHO with the deliberate intention of replacing the word monkeypox, to counter racial and geographical stigmatization and discrimination.

While rewording of this sort is challenging enough, it has proved even harder to reword or even give expression to descriptions of the behaviours and activities associated with mpox transmission.

“We encourage the use of clear, simple, descriptive and non-judgmental language when talking about mpox and how it spreads, to improve the chances of people understanding and therefore being able to manage their own risks,” says Lewis. “However, it has not always been easy to meet that standard, partly because of people’s reluctance to get into the details.”

Doherty has similar concerns. “If you look at the WHO mpox website, mpox is defined as a viral infection which can spread between people, mainly through close contact, and occasionally from the environment to people via things and surfaces,” she says. 

As to why euphemism seems to have such a hold on mpox guidance and broader mpox discourse, Lewis suggests that sociocultural biases and taboos may be at work. “It’s also possible that people, including people within WHO, avoid talking explicitly about the activities of certain groups because they fear a backlash against the communities they are seeking to inform, especially in countries where homosexuality is illegal or sanctioned in some way,” she adds.

In other words, there may be a perception in some sectors of the public health community that broadly disseminated explicit information is unhelpful. “There is clearly a challenge here,” says Lewis. “How do you disseminate the information communities need without driving stigma?”

Is more targeted messaging the answer? Dawie Nel thinks so. The director of OUT, an organization focused on the health and rights of LGBTQ+ individuals in South Africa, Nel advocates for targeted messaging that reflects realities on the ground.

“We enter the networks.”

Dawie Nel

South Africa’s mpox epidemic has so far been relatively small, but has drawn attention because of the severity of its 25 recorded cases. “The current outbreak appears to be driven by local transmission, as all cases have occurred among men who have sex with men with no international travel history,” says Joseph Adams, OUT’s programme manager.

Most of those infected were reported to be individuals with unmanaged or advanced HIV disease, increasing their susceptibility to extensive skin lesions and other medical complications. Almost all the men have required hospitalization. Adams believes there are probably more cases going unreported. 

Like Nel, Adams thinks the government could be doing more to target and clarify its messaging. “There is insufficient information for key populations,” he says, “and the messaging tends to be too vague or seems out of touch with reality on the ground.”

Nel believes that to get the reality on the ground it is necessary to engage with the highest-risk communities over extended periods. “We don’t just show up at brothels or bars, we enter the networks,” he says.

In Soweto, for example, OUT works with a group of 120 Chemsex MSM (men who use drugs to enhance and prolong sexual experiences). “We were on the ground for three years before we started to have an impact,” says Nel. “Such engagement not only helps build trust, it makes it possible to identify people who have influence in the community.”

Furaha also questions the effectiveness of vague public announcements, while acknowledging that government and partners are using a range of channels including radio and messaging services. “We go around the city at night to the bars, and we spell out the risks to the women and we distribute condoms,” she says.

However, unlike Nel, Furaha does not have the luxury of the protracted engagement needed to build trust. “One of the challenges we face is that it is always a new set of faces,” she says. Nor can she hope for much change in behaviours, other than an increased use of condoms, more careful choice of clients and greater use of the limited health facilities available. 

She would also encourage the women to get vaccinated if that were an option. Soon it may be. With support from WHO and other partners, the Ministry of Public Health, Hygiene and Social Protection launched their first mpox vaccination campaign in October. “For the first time, the vulnerable groups to benefit explicitly include key populations such as sex workers and men who have sex with men,” says Lewis. “With thoughtful planning and careful implementation that ensures delivery of stigma-free health services, this could be a game-changer.”

**Figure:**
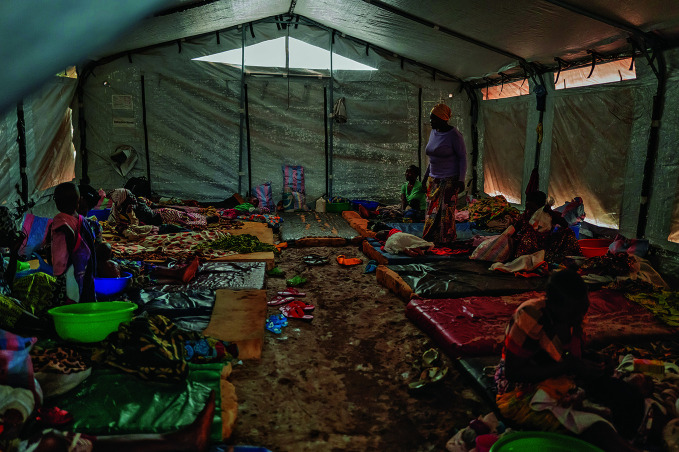
Female mpox patients in Kavumu Hospital in South Kivu, Democratic Republic of the Congo.

WHO/Guerchom Ndebo

**Figure:**
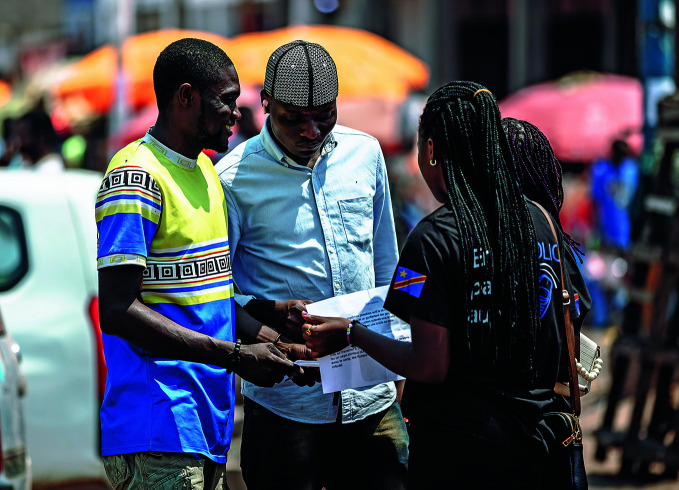
Raising awareness about mpox in Kalemie, Tanganyika province, Democratic Republic of the Congo.

UNICEF/Ibrahim Kalombo Rivild

